# The Role of Geometrically Necessary Dislocations in Cantilever Beam Bending Experiments of Single Crystals

**DOI:** 10.3390/ma10030289

**Published:** 2017-03-16

**Authors:** Edgar Husser, Swantje Bargmann

**Affiliations:** 1 Institute of Continuum Mechanics and Material Mechanics, Hamburg University of Technology, 21073 Hamburg, Germany; 2 Institute of Materials Research, Materials Mechanics, Helmholtz-Zentrum Geesthacht, 21502 Geesthacht, Germany; 3 Chair of Solid Mechanics, School of Mechanical Engineering and Safety Engineering, University of Wuppertal, 42119 Wuppertal, Germany; bargmann@uni-wuppertal.de

**Keywords:** cantilever beam bending, size effect, geometrically necessary dislocations, crystal plasticity, finite element method

## Abstract

The mechanical behavior of single crystalline, micro-sized copper is investigated in the context of cantilever beam bending experiments. Particular focus is on the role of geometrically necessary dislocations (GNDs) during bending-dominated load conditions and their impact on the characteristic bending size effect. Three different sample sizes are considered in this work with main variation in thickness. A gradient extended crystal plasticity model is presented and applied in a three-dimensional finite-element (FE) framework considering slip system-based edge and screw components of the dislocation density vector. The underlying mathematical model contains non-standard evolution equations for GNDs, crystal-specific interaction relations, and higher-order boundary conditions. Moreover, two element formulations are examined and compared with respect to size-independent as well as size-dependent bending behavior. The first formulation is based on a linear interpolation of the displacement and the GND density field together with a full integration scheme whereas the second is based on a mixed interpolation scheme. While the GND density fields are treated equivalently, the displacement field is interpolated quadratically in combination with a reduced integration scheme. Computational results indicate that GND storage in small cantilever beams strongly influences the evolution of statistically stored dislocations (SSDs) and, hence, the distribution of the total dislocation density. As a particular example, the mechanical bending behavior in the case of a physically motivated limitation of GND storage is studied. The resulting impact on the mechanical bending response as well as on the predicted size effect is analyzed. Obtained results are discussed and related to experimental findings from the literature.

## 1. Introduction

Micromechanical testing of small-scaled single crystals has been excessively practiced in the last two decades to study the mechanical size-dependence of diverse materials [1,2,3,4]. Different intrinsic (microstructural) effects have been found to be triggered by the interplay of physical size limitation such as free surfaces and the underlying microstructure in which the initial density of dislocations plays a crucial role. For example, experiments and 3D-discrete dislocation dynamics (DDD) simulations indicated that the size-dependent response of Ni single crystals decreases with increasing starting dislocation density [5]. Further, stable plastic deformation for crystals of ∼360 nm size was achieved and no strengthening effect in the range of 360 nm–1500 nm was observed for Mo alloy microcrystals [6]. In both cases, the crystals were machined from relatively strong pre-strained bulk. Conversely, a sufficiently low initial dislocation density provokes a rapid starvation of available dislocations [7]. However, this effect becomes dominant if, for instance, the diameter *D* of micropillar samples falls below a critical value (∼1 μm in [7]). In the study of Shan et al. [8], an initial dislocation density of ∼$10^{15}$ m^−2^ has fallen very fast to zero for D=160 nm whereas already larger samples (D≥250 nm) were less likely to be dislocation-free after testing. Besides the characteristic sample dimension, the overall crystal size may additionally be considered as suggested recently by El-Awady [9].

With increasing sample size, other mechanisms will superimpose and start to affect or even govern the mechanical behavior. Depending on the geometry of deformation induced into the sample in terms of the applied loading, plastic strain gradients and associated geometrically necessary dislocations therewith, may increase significantly the plastic work hardening. Bending represents a typical deformation mode in which plastic strain gradients are induced extrinsically. Fleck and Hutchinson [10] suggest that the stored density of GNDs is proportional to the curvature of the bended beam. Experiments have found a strong inverse correlation between beam thickness and increase in strength of the material. For instance, an increase in the work hardening behavior with decreasing beam thickness has been reported by Stölken and Evans [11] for thin beams with a variation in thickness between 12.5 μm and 100 μm. Microbending experiments of Motz et al. [12] confirmed the strong correlation between flow stress and beam thickness. The investigated beam sizes varied in thickness between 7.5 μm and 1 μm. In the same work, it was pointed out that a regular alignment of GNDs (as would be expected in pure bending) is insufficient to explain the size effect. The responsible mechanism could be dislocation source limitation as a result of a rapid starvation of initially available dislocations as well as back-stress effects induced by dislocation pile-ups along the neutral axis. Numerical results of 3D-DDD beam bending simulations with thicknesses between 0.5 μm and 1.5 μm revealed that a combination of pile-up of GNDs and source size limitation mimics the experimental data quiet well [13]. These findings indicate the complexity of size-dependent strengthening behavior due to superposition of different mechanisms. Accordingly, it is difficult to independently estimate the size-dependent hardening contribution associated with each mechanism based on experimental data. For that reason, a gradient extended crystal plasticity model is used to investigate the size range (characterized by means of the beam thickness) and the extent to which GNDs affect the size-dependent bending behavior of small-scaled cantilever beams.

The aim of this work is to examine the size-dependent bending response for a range of sample sizes. In particular, we focus on three sample sizes with a variation in thickness between 2.5 μm and 5.0 μm. For this purpose, a higher-order gradient crystal plasticity model is implemented in a three-dimensional finite element framework. Highlights of the model are non-standard evolution relations for edge and screw components of the slip system-based dislocation density vector, crystal-specific interaction relations, and higher-order gradient boundary conditions. The gradient effect associated with accumulating GND densities is of primary interest. In order to gain additional insights, the size-dependent impact of GNDs on the evolution of SSDs is investigated as well. The mechanical response of cantilever beams is further addressed for the case when the evolution of GNDs saturates due to a physical limitation. The number of dislocations to be stored locally due to compatibility reasons cannot be arbitrary high. Accordingly, a feasible limit for GND storage is introduced. All results are discussed and related to experimental findings from the literature.

## 2. Model Description

### 2.1. Higher-Order Gradient Crystal Plasticity Theory

The large deformation-based, gradient-enhanced crystal plasticity theory of Bargmann et al. [14] is followed, see also [15]. As part of this, the multiplicative decomposition of the deformation gradient $F=F_{E}\cdot F_{P}$ into an elastic $F_{E}$ and a plastic part $F_{P}$ is adopted. The state space is specified by $s=(C_{E},\left\{\gamma_{\alpha }\right\},\left\{\nabla_{i}\gamma_{\alpha }\right\})$, in which $C_{E}=F_{E}^{T}\cdot F_{E}$ denotes the elastic right Cauchy–Green strain tensor, $\left\{\gamma_{\alpha }\right\}$ represents a set of plastic slip variables, and $\left\{\nabla_{i}\gamma_{\alpha }\right\}$ is the corresponding set of plastic slip gradients. Both sets scale with the number of admitted slip systems, where *α* denotes a particular slip system. In this framework, the plastic slip $\gamma_{\alpha }$ is used as a primary measure for plastic deformation

(1) $$\begin{array}{c} \gamma_{\alpha }=\int \left|\nu_{\alpha }\right|\mathrm{dt}. \end{array}$$

Although the plastic slip rate $\nu_{\alpha }$ may not necessarily represent the material-time derivative of $\gamma_{\alpha }$, such an approximation is often applied in practice. This assumption is adopted in the following.

We employ a thermodynamic consistent formulation in which the free energy storage with respect to the intermediate configuration is expressed by the following additive split

(2) $$\begin{array}{cc} \psi_{i} & =\psi_{i}\left(C_{E},\left\{\gamma_{\alpha }\right\},\left\{\nabla_{i}\gamma_{\alpha }\right\}\right)\\ & =\psi_{i}^{e}\left(C_{E}\right)+\psi_{i}^{l}\left(\left\{\gamma_{\alpha }\right\}\right)+\psi_{i}^{g}\left(\left\{\nabla_{i}\gamma_{\alpha }\right\}\right), \end{array}$$

such that $\psi_{i}^{e}\left(C_{E}\right)$ represents a hyperelastic contribution, $\psi_{i}^{l}\left(\left\{\gamma_{\alpha }\right\}\right)$ covers the local energy storage due to dislocation glide, and $\psi_{i}^{g}\left(\left\{\nabla_{i}\gamma_{\alpha }\right\}\right)$ is the gradient related energy storage due to accumulation of GNDs. The affiliation of a physical quantity • is indicated by the following notation: ${•}_{r}$ (reference space), ${•}_{i}$ (intermediate space), ${•}_{c}$ (current space). This notation is also applied to differential operators, e.g., $\nabla_{i}•$, ${\mathrm{Div}}_{i}\left(•\right)$, and ${\mathrm{Curl}}_{i}\left(•\right)$.

Under quasi-static, isothermal conditions, the dissipation inequality with respect to the intermediate configuration reads

(3) $$\begin{array}{c} D=\int_{B_{i}}\left[\rho_{i}P-\Upsilon_{i}(\dot{C_{E}},\left\{\nu_{\alpha }\right\},\left\{\nabla_{i}\nu_{\alpha }\right\}\right]{\mathrm{dV}}_{i}\geq 0. \end{array}$$

In this, the stress power density $P=P_{E}+P_{P}$ is decomposed into an elastic part $P_{E}=1/\left[2\rho_{i}\right]S_{E}:{\dot{C}}_{E}$, based on the second Piola–Kirchhoff stress tensor $S_{E}$, and a plastic part $P_{P}=1/\rho_{i}M_{E}:L_{P}$, in which $M_{E}=C_{E}\cdot S_{E}$ is the Mandel stress tensor and $L_{P}={\dot{F}}_{P}\cdot F_{P}^{-1}=\sum_{\alpha }\nu_{\alpha }\left[s_{\alpha }⊗n_{\alpha }\right]$ denotes the plastic velocity gradient defined as a superposition of individual plastic slip rates on corresponding slip systems. As usual, $s_{\alpha }$, $n_{\alpha }$, and $t_{\alpha }=n_{\alpha }\times s_{\alpha }$ are the slip direction, slip plane normal, and transverse direction vectors respectively. The intermediate configuration is assumed to be isoclinic (Any set of orthonormal vectors $\left\{s_{\alpha },t_{\alpha },n_{\alpha }\right\}$ which describes the crystallographic geometry of a slip system does not change between the reference and the intermediate configuration [16]. Hence plastic deformation is lattice (volume) preserving, i.e., $\det(F_{P})=1$.). Further, $\Upsilon_{i}$ represents the specific energy storage rate

(4) $$\begin{array}{c} \Upsilon_{i}=\frac{\partial \psi_{i}^{e}}{\partial C_{E}}:\dot{C_{E}}+\sum_{\alpha }\left[\frac{\partial \psi_{i}^{l}}{\partial \gamma_{\alpha }}\nu_{\alpha }+\frac{\partial \psi_{i}^{g}}{\partial \nabla_{i}\gamma_{\alpha }}\cdot \nabla_{i}\nu_{\alpha }\right]. \end{array}$$

Following standard thermodynamic arguments, the constitutive relation for the hyperelastic state results in $S_{E}=2\;\partial \psi_{i}^{e}/\partial C_{E}$. In accordance to Ekh et al. [17], we introduce the scalar-valued micro-stress $\kappa_{\alpha }=\partial \psi_{i}^{l}/\partial \gamma_{\alpha }$ and the back-stress vector (or vector-valued micro-stress) $\kappa_{\alpha }=\partial \psi_{i}^{g}/\partial \nabla_{i}\gamma_{\alpha }$ work conjugate to $\gamma_{\alpha }$ and $\nabla_{i}\gamma_{\alpha }$ respectively. Substituting these constitutive relations into Equation (3) leads to the reduced dissipation inequality

(5) Dred=∫BiME:LP−ab∑ακανα+κα·∇iναdVi≥0.

Integration by parts and applying Gauss’s theorem gives

(6) $$\begin{array}{cc} D_{\mathrm{red}} & =\int_{B_{i}}\left[M_{E}:L_{P}-\sum_{\alpha }\left[\kappa_{\alpha }-{\mathrm{Div}}_{i}\left(\kappa_{\alpha }\right)\right]\nu_{\alpha }\right]{\mathrm{dV}}_{i}-\int_{\partial B_{i}}\sum_{\alpha }\nu_{\alpha }\;\kappa_{\alpha }\cdot N_{i}^{\left(b\right)}{\mathrm{dA}}_{i}\geq 0. \end{array}$$

A more restrictive validity of the dissipation inequality is obtained by its local form. This gives with respect to the bulk dissipation

(7) $$\begin{array}{cc} D_{\mathrm{red}}^{L} & =M_{E}:L_{P}-\sum_{\alpha }\left[\kappa_{\alpha }-{\mathrm{Div}}_{i}\left(\kappa_{\alpha }\right)\right]\nu_{\alpha }=\sum_{\alpha }\left[\tau_{\alpha }-\kappa_{\alpha }+{\mathrm{Div}}_{i}\left(\kappa_{\alpha }\right)\right]\nu_{\alpha }\geq 0, \end{array}$$

and with respect to the boundary dissipation

(8) $$\begin{array}{c} D_{\mathrm{red}}^{L(b)}=-\sum_{\alpha }\nu_{\alpha }\;\kappa_{\alpha }\cdot N_{i}^{\left(b\right)}=\sum_{\alpha }\nu_{\alpha }\;\kappa_{\alpha }^{\left(b\right)}\geq 0, \end{array}$$

where $\tau_{\alpha }=M_{E}:\left[s_{\alpha }⊗n_{\alpha }\right]$ represents the resolved shear stress and $\kappa_{\alpha }^{\left(b\right)}=-\kappa_{\alpha }\cdot N_{i}^{\left(b\right)}$ denotes the micro-hardening stress projected on the outward pointing normal vector $N_{i}^{\left(b\right)}$—here associated with an infinitesimal intermediate surface element. The relation between the referential surface normal vector and the intermediate surface normal vector is given by the cofactor of the plastic deformation, i.e., $N_{i}\;{\mathrm{dA}}_{i}=\mathrm{cof}\left(F_{P}\right)N_{r}{\mathrm{dA}}_{r}$. The current rate of dissipating work depends on the history of plastic deformation in terms of $\gamma_{\alpha }$ but also on the current plastic slip rate $\nu_{\alpha }$, cf. Equations (7) and (8). Moreover, for homogeneous plastic deformation, i.e., $\kappa_{\alpha }=0$, Equation (7) reduces to $D_{\mathrm{red}}^{L}=\sum_{\alpha }\left[\tau_{\alpha }-\kappa_{\alpha }\right]\nu_{\alpha }\geq 0$ whereas the boundary contribution in Equation (8) completely vanishes.

Then, the onset of plastic yielding is regulated by means of a yield function $\phi_{\alpha }$ for each slip system, here defined as

(9) $$\begin{array}{c} \phi_{\alpha }=\tau_{\alpha }-\kappa_{\alpha }+{\mathrm{Div}}_{i}\left(\kappa_{\alpha }\right)-Y_{\alpha }=\tau_{\alpha }-S_{\alpha }, \end{array}$$

where $Y_{\alpha }$ defines the initiation of plastic yield in the absence of hardening, i.e., the initial slip system resistance which is equivalent to the critical resolved shear stress of the system, whereas $S_{\alpha }=\kappa_{\alpha }-{\mathrm{Div}}_{i}\left(\kappa_{\alpha }\right)+Y_{\alpha }$ is the current slip system resistance.

In the context of viscoplasticity, overstress states $\tau_{\alpha }-S_{\alpha }>0$ are generally allowed and typically regularized via a power law relation. Here, a Perzyna form is chosen as a viscoplastic regularization:

(10) $$\begin{array}{c} \nu_{\alpha }=\nu_{0}{\left[\frac{<\phi_{\alpha }>}{C_{0}}\right]}^{m}. \end{array}$$

The brackets define a ramp function of the form $<\phi_{\alpha }>=1/2\;\left[\phi_{\alpha }\;+\left|\phi_{\alpha }\right|\right]$ and ensure that $\nu_{\alpha }\neq 0$ only for $\tau_{\alpha }>S_{\alpha }$. Moreover, *m* denotes the rate sensitivity exponent of the stress ratio, $\nu_{0}$ represents the reference shear rate, and $C_{0}$ is the drag stress.

### 2.2. Governing Equations

The so-called dislocation tensor represents a continuum measurement for GND densities from which the vector-valued GND vector ${\dot{g}}_{i\alpha }$ is derived, cf. [18]. In extension to [14,], the evolution of ${\dot{g}}_{i\alpha }$—here with respect to the intermediate configuration—is taken as

(11) $$\begin{array}{cc} {\dot{g}}_{i\alpha } & =\sum_{\beta }\left[\nu_{\alpha }\left[n_{\alpha }\cdot s_{\beta }\right]g_{i\beta }+\nu_{\beta }\left[n_{\alpha }\cdot g_{i\alpha }\right]s_{\beta }\right]+|b_{\alpha }{|}^{-1}\nabla_{i}\nu_{\alpha }\times n_{\alpha }, \end{array}$$

where $b_{\alpha }$ denotes the Burgers vector. This relation accounts for dislocation collision processes, for instance, interactions between mobile dislocations of active slip systems mimicked by the plastic slip rates and stored dislocations on latent slip systems. For most applications, it is sufficient to account for the edge and screw component of the GND density vector. It is assumed that there is no development of plastic slip gradients perpendicular to the slip planes, i.e., ${\dot{g}}_{i\alpha }\times n_{\alpha }=0$[19]. With respect to fcc crystals, modified interaction relations are proposed for the edge and screw components of the GND density based on moduli for edge–edge $\iota_{\alpha \beta }^{ee}$, edge–screw $\iota_{\alpha \beta }^{es}$, screw–screw $\iota_{\alpha \beta }^{ss}$, and screw–edge $\iota_{\alpha \beta }^{se}$ dislocation intersections:

(12) $$\begin{array}{cc} \iota_{\alpha \beta }^{ee} & =\left|s_{\alpha }\cdot s_{\beta }\right|\left|n_{\alpha }\times n_{\beta }\right|,\\ \iota_{\alpha \beta }^{es} & =\left|s_{\alpha }\cdot t_{\beta }\right|\left|n_{\alpha }\times n_{\beta }\right|,\\ \iota_{\alpha \beta }^{ss} & =\left|t_{\alpha }\cdot t_{\beta }\right|\left|n_{\alpha }\times n_{\beta }\right|,\\ \iota_{\alpha \beta }^{se} & =\left|t_{\alpha }\cdot s_{\beta }\right|\left|n_{\alpha }\times n_{\beta }\right|. \end{array}$$

In addition, slip coplanarity moduli $\chi_{\alpha \beta }$ are introduced in accordance to previous works (e.g., [20,21,22]) as

(13) $$\begin{array}{c} \chi_{\alpha \beta }=\left\{\begin{array}{c} 0\;\mathrm{for}\;\left|n_{\alpha }\times n_{\beta }\right|\neq 0\;\mathrm{noncoplanar},\\ 1\;\mathrm{for}\;\left|n_{\alpha }\times n_{\beta }\right|=0\;\mathrm{coplanar}. \end{array}\right. \end{array}$$

In the case of coplanar slip systems, i.e., slip planes of system *α* and *β* are parallel to each other, all intersection moduli vanish and slip-system interactions are solely determined by coplanarity moduli. Recall that the GND components are obtained by projecting the plastic slip gradients on the slip directions $s_{\alpha }$ and the transverse slip directions $t_{\alpha }$ (see Arsenlis and Parks [23], Gurtin and Anand [24]). Then, under consideration of the above introduced interaction moduli, Equation (11) is substituted by the following two scalar-valued field equations which read

(14) $$\begin{array}{cc} {\dot{g}}_{i\alpha }^{e} & =\sum_{\beta }\nu_{\alpha }\left[g_{i\beta }^{e}\left[\iota_{\alpha \beta }^{ee}+\chi_{\alpha \beta }\right]+g_{i\beta }^{s}\left[\iota_{\alpha \beta }^{es}+\chi_{\alpha \beta }\right]\right]-{\left|b_{\alpha }\right|}^{-1}\nabla_{i}\nu_{\alpha }\cdot s_{\alpha } \end{array}$$

and

(15) $$\begin{array}{cc} {\dot{g}}_{i\alpha }^{s} & =\sum_{\beta }\nu_{\alpha }\left[g_{i\beta }^{s}\left[\iota_{\alpha \beta }^{ss}+\chi_{\alpha \beta }\right]+g_{i\beta }^{e}\left[\iota_{\alpha \beta }^{se}+\chi_{\alpha \beta }\right]\right]-{\left|b_{\alpha }\right|}^{-1}\nabla_{i}\nu_{\alpha }\cdot t_{\alpha }, \end{array}$$

respectively. The first relation in Equation (14) accounts for the impact of stored edge and screw GND densities (with respect to latent slip systems *β*) on the evolution of the edge GND density of slip system *α* whereas the second term measures the geometrically necessary edge dislocation density by means of the plastic slip gradient $\nabla_{i}\nu_{\alpha }$. A similar relation is given for the geometrically necessary screw dislocation density. The negative sign of the second term in Equation (15) yields a convention in which the right-handed screw dislocation segment is treated as positive. An equivalent expression is found in the literature where a positive sign is used together with the line direction vector $l_{\alpha }=-t_{\alpha }$.

For the sake of completeness, we recall the balance of linear momentum for quasi-static and isothermal conditions and in the absence of body forces

(16) $$\begin{array}{c} 0={\mathrm{Div}}_{i}\left(F_{E}\cdot S_{E}\right). \end{array}$$

where $P=F_{E}\cdot S_{E}\cdot F_{P}^{-T}$ is the relation between the first and second Piola–Kirchhoff stress tensor.

### 2.3. Constitutive Relations

The hyperelastic energy contribution is postulated in terms of the Neo–Hookean law

(17) $$\begin{array}{c} \psi_{i}^{e}=\frac{1}{2}\mu \left[I_{C_{E}}-3\right]+\frac{1}{2}\lambda \ln{\left(J_{E}\right)}^{2}-\mu \ln\left(J_{E}\right), \end{array}$$

where $J_{E}=\det\left(F_{E}\right)=\det{\left(C_{E}\right)}^{1/2}$ is the elastic Jacobian determinant, $I_{C_{E}}=\mathrm{tr}\left(C_{E}\right)$ the first invariant of $C_{E}$, and *μ* together with *λ* denote the Lamé parameters. Then, the hyperelastic stress response results in

(18) $$\begin{array}{c} S_{E}=2\frac{\partial \psi_{i}}{\partial C_{E}}=\mu I+\left[\lambda \ln(J_{E})-\mu \right]C_{E}^{-1}. \end{array}$$

As observed in various investigations (cf. [12,25,26]), the stress–strain response of copper single crystal is characterized by a pronounced saturation behavior. In the study of Kleemola and Nieminen [27], it was found that Voce hardening [28] represents the best choice to describe the hardening behavior of Cu crystals. Therefore, the local energy contribution associated with dislocation glide (see [29]) reads

(19) ψil=∑αγαΔHαlabb+ΔHαlcsatexp−csat∑βγβ−1.

$c_{\mathrm{sat}}$ is the saturation rate parameter and $\Delta H_{\alpha }^{l}=H_{0}^{l}-Y_{\alpha }$ is the saturation hardening defined as the difference between local hardening modulus $H_{0}^{l}$ and initial yield resistance $Y_{\alpha }$ of the slip system. In this hardening law, latent hardening is addressed in terms of the accumulated plastic slip $\sum_{\beta }\gamma_{\beta }$ within the exponential function such that high slip system activity increases the effective local hardening contribution. The corresponding scalar-valued micro-stress $\kappa_{\alpha }^{l}$ is derived as

(20) $$\begin{array}{c} \kappa_{\alpha }^{l}=\frac{\partial \psi_{i}^{l}}{\partial \gamma_{\alpha }}=\Delta H_{\alpha }^{l}\left[1-\exp\left(-c_{\mathrm{sat}}\;\sum_{\beta }\gamma_{\beta }\right)\right]. \end{array}$$

Recalling that the free energy increases due to storage of GNDs in the material, edge and screw dislocation characters associated with distortion and twisting of the crystal lattice respectively, are introduced into the defect energy by the form

(21) $$\begin{array}{cc} \psi_{i}^{g} & =\frac{1}{2}\sum_{\alpha }l_{\alpha }^{2}{\left|b_{\alpha }\right|}^{2}\left[H_{0}^{e}{\left[g_{i\alpha }^{e}\right]}^{2}+H_{0}^{s}{\left[g_{i\alpha }^{s}\right]}^{2}\right]. \end{array}$$

$H_{0}^{e}$ and $H_{0}^{s}$ are the gradient hardening moduli related to the edge and screw component respectively, and $l_{\alpha }$ is a constitutive internal length scale parameter. The relation between the gradient hardening moduli is taken as

(22) $$\begin{array}{c} H_{0}^{s}=H_{0}^{e}\;\left[1-\nu \right], \end{array}$$

according to their elastic strain energy ratio (cf. [30]) where ν=λ/[2λ+2μ] is the Poisson’s ratio. Then, the constitutive relation for the back-stress vector is obtained from the chain rule

(23) $$\begin{array}{cc} \kappa_{\alpha } & =\frac{\partial \psi_{i}^{g}}{\partial \nabla_{i}\gamma_{\alpha }}=\frac{\partial \psi_{i}^{g}}{\partial g_{i\alpha }^{e}}\frac{\partial g_{i\alpha }^{e}}{\partial \nabla_{i}\gamma_{\alpha }}+\frac{\partial \psi_{i}^{g}}{\partial g_{i\alpha }^{s}}\frac{\partial g_{i\alpha }^{s}}{\partial \nabla_{i}\gamma_{\alpha }}. \end{array}$$

Finally, due to the dependence of ${\dot{g}}_{i\alpha }^{e}$ and ${\dot{g}}_{i\alpha }^{e}$ on $\nu_{\alpha }$, the scalar-valued micro-stress $\kappa_{\alpha }^{g}$ related to interaction processes is introduced via the relation

(24) $$\begin{array}{cc} \kappa_{\alpha }^{g} & =\frac{\partial \psi_{i}^{g}}{\partial \gamma_{\alpha }}=\frac{\partial \psi_{i}^{g}}{\partial g_{i\alpha }^{e}}\frac{\partial g_{i\alpha }^{e}}{\partial \gamma_{\alpha }}+\frac{\partial \psi_{i}^{g}}{\partial g_{i\alpha }^{s}}\frac{\partial g_{i\alpha }^{s}}{\partial \gamma_{\alpha }}. \end{array}$$

In fact, $\kappa_{\alpha }^{g}$ describes a non-local hardening contribution based on GND intersection and collision effects which are particularly pronounced upon load reversal. In the end, the micro-stress $\kappa_{\alpha }$ is comprised of a local as well as a non-local contribution, i.e., $\kappa_{\alpha }=\kappa_{\alpha }^{l}+\kappa_{\alpha }^{g}$.

### 2.4. Boundary Conditions

As we study the bending behavior of a single crystal with free surfaces, microfree boundaries are chosen. They refuse any dislocation pile-ups at the exterior of the crystal and, hence, boundaries appear to be transparent to dislocation motion

(25) $$\begin{array}{c} {\dot{g}}_{i}^{e}={\dot{g}}_{i}^{s}=0\;\mathrm{on}\;\partial B_{i}^{g}. \end{array}$$

This results in zero boundary dissipation according to Equation (8). An alternative approach based on non-idealized boundary conditions with non-zero boundary dissipation involving the effect of boundary yielding is presented in Husser et al. [30].

## 3. Numerical Implementation

The solution algorithm for the highly coupled and strongly non-linear multi-field problem is based on the dual-mixed finite element method as proposed by Ekh et al. [17]; see also Bargmann et al. [14,31]. In this, GND densities are introduced as nodal degrees of freedom in addition to the displacement. The basis for implementing the material model into a finite element framework is the variational form of the underlying governing equations. Applying the principle of virtual work to Equation (16) yields the variational form

(26) $$\begin{array}{c} 0=\int_{B_{i}}\frac{1}{2}S_{E}:\delta C_{E}{\mathrm{dV}}_{i}-\int_{\partial B_{i}}\delta u\cdot \left[F_{E}\cdot S_{E}\right]\cdot N_{i}^{\left(b\right)}{\mathrm{dA}}_{i}, \end{array}$$

where δu is a vector-valued test function and $\delta C_{E}=\left[F_{E}^{T}\cdot \nabla_{i}\left(\delta u\right)+\nabla_{i}{\left(\delta u\right)}^{T}\cdot F_{E}\right]$ denotes the variation of the right Cauchy–Green strain tensor. The corresponding mechanical boundary conditions read

(27) $$\begin{array}{cc} F_{E}\cdot S_{E}\cdot N_{i}^{\left(b\right)} & =0\;\mathrm{on}\;\partial B_{i}^{F}\\ \mathrm{and} & \\ u & =\bar{u}\;\mathrm{on}\;\partial B_{i}^{u}. \end{array}$$

In a similar manner, the variational forms of Equations (14) and (15) are obtained. We further choose an implicit finite-difference method for the time discretization of the global field relations such that ${\dot{g}}_{i\alpha }^{e}=\Delta g_{i\alpha }^{e}/\Delta t$, where Δt measures the current time increment and $\Delta g_{i\alpha }^{e}=g_{i\alpha (n+1)}^{e}-g_{i\alpha (n)}^{e}$. The same holds for the screw component, i.e., $\Delta g_{i\alpha }^{s}=g_{i\alpha (n+1)}^{s}-g_{i\alpha (n)}^{s}$. As an approximation, plastic slip rates are discretized analogically, i.e., $\nu_{\alpha }=\Delta \gamma_{\alpha }/\Delta t$, where $\Delta \gamma_{\alpha }=\gamma_{\alpha (n+1)}-\gamma_{\alpha (n)}$. With this at hand, the variational forms are written as

(28) $$\begin{array}{cc} 0 & =\int_{B_{i}}\delta g_{\alpha }^{e}\Delta g_{i\alpha }^{e}{\mathrm{dV}}_{i}-\int_{B_{i}}\delta g_{\alpha }^{e}\sum_{\beta }\Delta \gamma_{\alpha }\left[g_{i\beta }^{e}\left[\iota_{\alpha \beta }^{ee}+\chi_{\alpha \beta }\right]+g_{i\beta }^{s}\left[\iota_{\alpha \beta }^{es}+\chi_{\alpha \beta }\right]\right]{\mathrm{dV}}_{i}\\ & -{\left|b_{\alpha }\right|}^{-1}\int_{B_{i}}\Delta \gamma_{\alpha }{\mathrm{Div}}_{i}\left(\delta g_{\alpha }^{e}s_{\alpha }\right){\mathrm{dV}}_{i}+{\left|b_{\alpha }\right|}^{-1}\int_{\partial B_{i}}\delta g_{\alpha }^{e}\Delta \gamma_{\alpha }N_{i}^{\left(b\right)}\cdot s_{\alpha }{\mathrm{dA}}_{i}, \end{array}$$

and

(29) $$\begin{array}{cc} 0 & =\int_{B_{i}}\delta g_{\alpha }^{s}\Delta g_{i\alpha }^{s}{\mathrm{dV}}_{i}-\int_{B_{i}}\delta g_{\alpha }^{s}\sum_{\beta }\Delta \gamma_{\alpha }\left[g_{i\beta }^{s}\left[\iota_{\alpha \beta }^{ss}+\chi_{\alpha \beta }\right]+g_{i\beta }^{e}\left[\iota_{\alpha \beta }^{se}+\chi_{\alpha \beta }\right]\right]{\mathrm{dV}}_{i}\\ & -{\left|b_{\alpha }\right|}^{-1}\int_{B_{i}}\Delta \gamma_{\alpha }{\mathrm{Div}}_{i}\left(\delta g_{\alpha }^{s}t_{\alpha }\right){\mathrm{dV}}_{i}+{\left|b_{\alpha }\right|}^{-1}\int_{\partial B_{i}}\delta g_{\alpha }^{s}\Delta \gamma_{\alpha }N_{i}^{\left(b\right)}\cdot t_{\alpha }{\mathrm{dA}}_{i}, \end{array}$$

respectively. Here, $\delta g_{\alpha }^{s}$ and $\delta g_{\alpha }^{e}$ are arbitrary test functions.

The time-discretized versions of the micro-stresses (Equations (23) and (24)) read

(30) $$\begin{array}{cc} \kappa_{\alpha } & =\frac{\partial \psi_{i(n+1)}^{g}}{\partial \nabla_{i}\gamma_{\alpha (n+1)}}=-l_{\alpha }^{2}\left|b_{\alpha }\right|H_{0}^{e}\left[g_{i\alpha }^{e}s_{\alpha }+\left[1-\nu \right]g_{i\alpha }^{s}t_{\alpha }\right] \end{array}$$

resp.

(31) $$\begin{array}{cc} \kappa_{\alpha }^{g}=\frac{\partial \psi_{i(n+1)}^{g}}{\partial \gamma_{\alpha (n+1)}} & =l_{\alpha }^{2}{\left|b_{\alpha }\right|}^{2}H_{0}^{e}\left[g_{i\alpha }^{e}\sum_{\beta }g_{i\beta }^{e}\left[\iota_{\alpha \beta }^{ee}+\chi_{\alpha \beta }\right]+g_{i\beta }^{s}\left[\iota_{\alpha \beta }^{es}+\chi_{\alpha \beta }\right]\right.\\ & +\left.\left[1-\nu \right]g_{i\alpha }^{s}\sum_{\beta }g_{i\beta }^{s}\left[\iota_{\alpha \beta }^{ss}+\chi_{\alpha \beta }\right]+g_{i\beta }^{e}\left[\iota_{\alpha \beta }^{se}+\chi_{\alpha \beta }\right]\right]. \end{array}$$

Both equations are fully implicit. Therefore, it is not explicitly indicated that the time-dependent quantities are associated with the new time increment $t_{\left(n+1\right)}=t_{\left(n\right)}+\Delta t$.

## 4. Set-Up of the Numerical Example

### 4.1. Finite Element Model

The presented gradient-based crystal plasticity model is applied to microbending experiments of copper single crystal. Three micron-sized cantilever beams with varying thickness *t* in the range between 2.5 μm and 5.0 μm are under investigation, see Table 1 for exact sample dimensions. In order to prevent additional influences on the size-dependent hardening, the momentum arm $l_{b}$ as well as the edge length in width direction *w* are kept constant for all geometries. This allows for a meaningful interpretation of the results for which a strong correlation between the strength of the material and the thickness of the beam is expected (Evans and Hutchinson [32]). Sample geometries and crystallographic orientation were exemplary chosen in accordance to the experimental set-up of Motz et al. [12]. Details regarding sample preparation and fabrication are provided in [33]. In that experimental study, various single crystalline cantilever beam samples were fabricated by the focused-ion beam (FIB) technique and loaded with an indenter tip at the free end.

**Figure 1:**
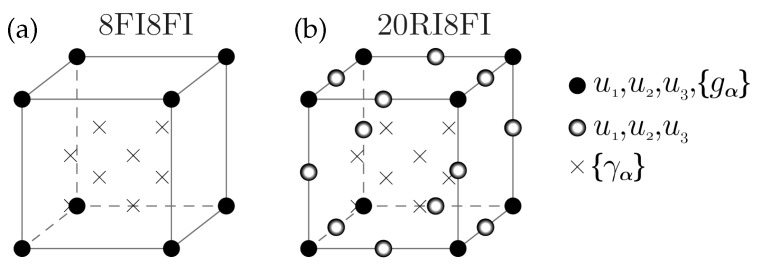
Schematic illustration of implemented finite element schemes: (**a**) Eight-node hexahedron element with full integration scheme (8FI8FI)—displacement fields and GND density fields are interpolated by trilinear shape functions; (**b**) Twenty-node brick element with mixed integration scheme (20RI8FI)—displacement fields are interpolated by quadratic serendipity shape functions combined with the reduced integration scheme whereas the GND density fields are interpolated by trilinear shape functions in combination with the full integration scheme.

**Figure 2:**
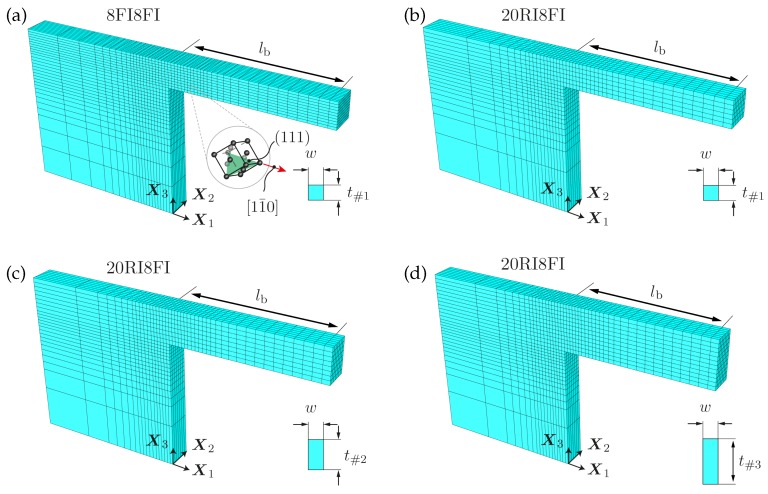
Finite-element meshes, crystallographic orientation, and dimensions of investigated cantilever beam geometries:(**a**,**b**) cantilever beam #1; (**c**) cantilever beam #2; (**d**) cantilever beam #3. Mesh (**a**) is generated by eight-node hexahedron elements whereas meshes (**b**–**d**) are generated by twenty-node brick elements, cf. Figure 1. Exact dimensions are summarized in Table 1.

With regard to the finite element model, quadratic serendipity elements (twenty-node) have been used for geometry approximation whereas displacement and the GND density degrees of freedom have been solved with a different number of nodes, cf. Figure 1. Here, the solution of the displacement field is based on a fully quadratic FE-approximation combined with a reduced integration scheme (2×2×2 Gauss points). This approach is known to be well suitable for bending-dominated problems. In contrast, the GND densities are only evaluated at the corner nodes in terms of a linear FE-approximation using the full integration scheme, see Figure 1b. This mixed-element formulation—henceforth denoted as 20RI8FI—was examined by Kuroda [34] for the two-dimensional case with respect to simple shear and compression problems and was revealed to be well suitable for applications in the context of higher-order gradient crystal plasticity as it exhibits a reliable performance. The finite element meshes of the cantilever beams are illustrated in Figure 2 whereas the corresponding geometry and discretization data is provided in Table 1. Figure 2 includes a mesh for which both fields are approximated with trilinear eight-node elements and 2×2×2 Gauss points (full integration scheme), cf. Figure 1a. In the results section, a comparison in performance between the mixed-element (20RI8FI) and the fully linear formulation (8FI8FI) is carried out for selected cases concerning cantilever beam #1.

### 4.2. Crystallography and Material

The crystallographic orientation of the crystals is chosen in accordance to the experiments in [12]: the $\left[1\bar{1}0\right]$ direction is aligned parallel to the longitudinal beam axis (parallel to $X_{1}$) and the (111)-plane is oriented parallel to the $X_{1}-X_{2}$ plane, cf. Figure 2a. The applied deflection results in the typical tensile and compressive dominated zones which determine the resolved shear stress on the individual slip systems and, thus, dominate their activation (at least for the here relevant bending load regime). There exist four non-zero Schmid factors $f_{\alpha }$ for the particular crystal orientation as indicated in Table 2 together with the slip system designation of the fcc lattice.

In the flow rule (see Equation (10)), each slip direction is associated with a stand-alone slip system. In fcc crystals, there are generally 12 edge and six screw dislocation characters as each screw dislocation line is shared by two slip planes, cf. [35].

The elasticity parameters for copper, i.e., Young’s modulus E=126.9 GPa and Poisson’s ratio ν=0.35, are taken from [12], respectively. Further, the initial yield limit is chosen to be $Y_{\alpha }=1.5$ MPa in agreement with experiments of single crystals, see for instance [36,37]. The magnitude of the Burgers vector is taken as $b=\left|b_{\alpha }\right|=a/\sqrt{2}=0.2552$ nm, based on a lattice constant of a=0.3609 nm [38]. In order to minimize rate effects, the rate-sensitivity parameter is chosen to be m=20 and the reference slip rate is put on a level with the macroscopic (quasi-static) load rate, i.e., $\nu_{0}={\dot{\varepsilon }}_{\mathrm{ben}}$. The drag stress parameter is assumed to be $C_{0}=10$ MPa. Moreover, the same constitutive length-scale parameter $l=l_{\alpha }$ is applied to all slip systems in analogy to the Burgers vector magnitude. In [11], *l* was found to be 4 μm for highly pure Ni. Since a similar magnitude was obtained for torsion tests of copper wires, cf. [39], this value is adopted here. The values for the hardening moduli and the saturation rate are discussed in Section 5.1. All material parameters are summarized in Table 3.

## 5. Numerical Results: Microbending of Cu Single Crystal

### 5.1. Element Choice: Eight-Node Hexahedron Element with Full Integration (8FI8FI) vs. Twenty-Node Brick Element with Mixed Integration (20RI8FI)

**Figure 3:**
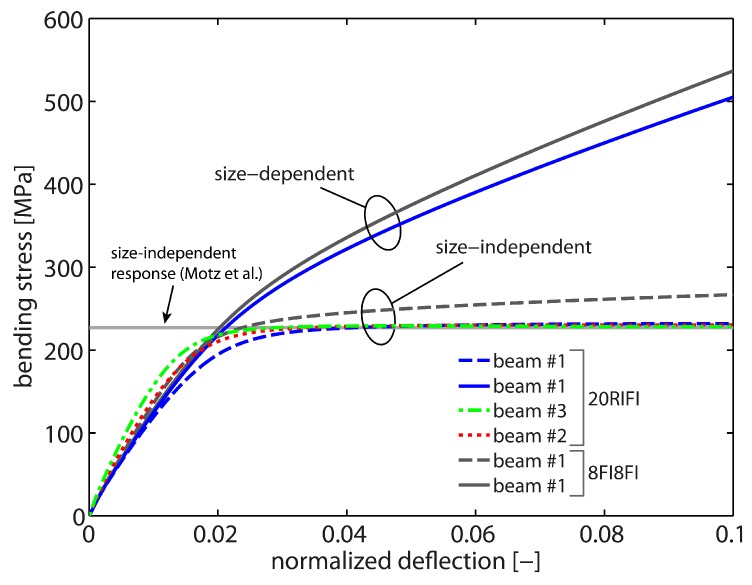
Size-independent bending response for $H_{0}^{l}=77.5$ MPa and $c_{\mathrm{sat}}=10^{3}$. Simulations with all three sample sizes (20RI8FI) yield the reference data of [12]. The FE-mesh with linear elements (8FI8FI) overestimates the stiffness as well as the strength due to volumetic locking in case of bending-dominated loading for the size-independent as well as the size-dependent case.

As a starting point, the size-independent response is studied, i.e., the bending response in the absence of gradient effects which is obtained when l/t≈0. By setting l=0, the computations mimic the response of bulk samples independent of the actual sample dimensions. A macroscopic bending test from [12] serves as a reference in order to calibrate the local hardening modulus $H_{0}^{l}$ as well as the saturation rate $c_{\mathrm{sat}}$. All numerical bending tests were loaded up to 10% normalized deflection (applied deflection/initial momentum arm). Optimal values are identified as $H_{0}^{l}=77.5$ MPa and $c_{\mathrm{sat}}=10^{3}$. The final results are shown in Figure 3. As seen, this choice results in a good saturation behavior with rapid hardening behavior for all three sample sizes associated with the 20RI8FI-formulation. All curves yield the reference flow stress of ≈227 MPa, i.e., the bending response is clearly size-independent (independent of the beam thickness).

By comparing the bending response of cantilever beam #1 of the two element formulations, it is clearly seen that the 8FI8FI-element formulation overestimates the strength and the bending stiffness. A deviation in the stress–strain curve is already obvious after ≈1% normalized deflection. Further, the saturation level as well as saturation behavior are not captured correctly. This is due to a bending-dominated deformation mode which cannot be properly captured by the linear element formulation due to locking effects.

Next, the size-dependent material behavior is investigated. Exemplarily, cantilever beam #1 is studied as the strongest impact is expected for the beam sample with the smallest thickness. A linear-like hardening behavior is observed (cf. Figure 3) which is associated with a continuously increasing (plastic) strain gradient during bending. The strain gradient scales the higher-order gradient hardening in terms of the back-stress ${\mathrm{Div}}_{i}\left(\kappa_{\alpha }\right)$. Hence, the gradient hardening contribution constantly increases which in the end prevents a saturation of the overall hardening response. As in the size-independent case, the 8FI8FI-formulation overestimates the bending response. Yet, the difference at the final deformation state appears to be not that pronounced which indicates that the computational accuracy of the GND field is not affected by the 8FI8FI-element formulation.

**Figure 4:**
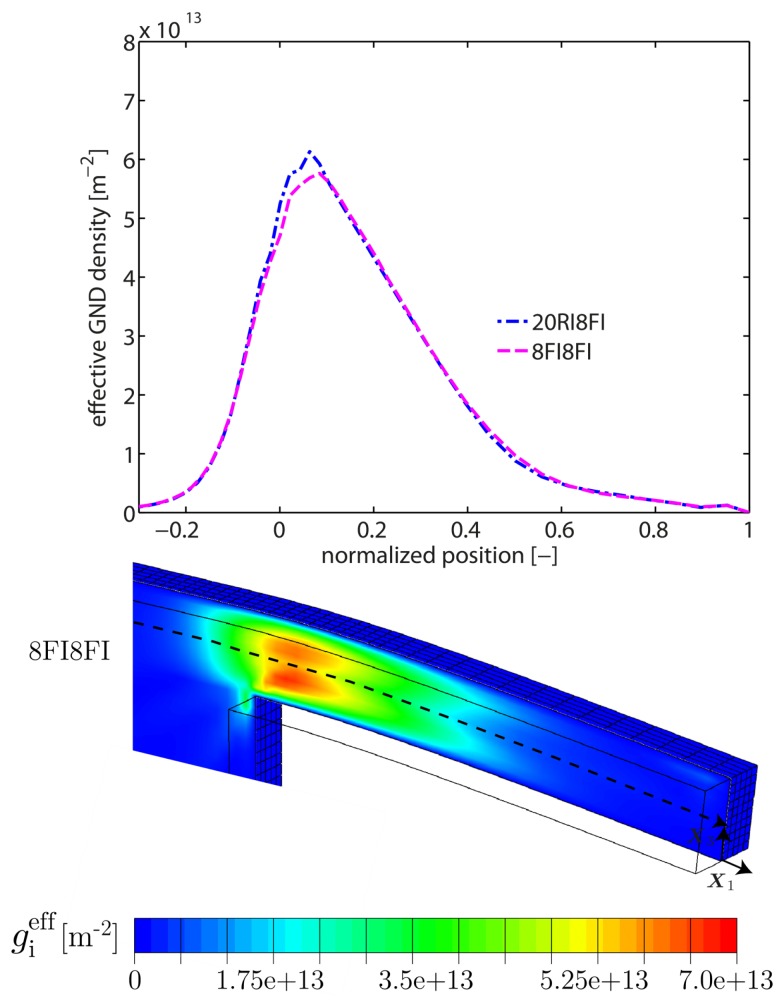
Distribution of the effective GND density $g_{i}^{\mathrm{eff}}$ for the cantilever beam sample #1 along the middle beam axis (from left to right) for the particular case $H_{0}^{e}=1$ GPa. The corresponding contour plot within the central $X_{1}-X_{3}$-plane is additionally shown for the 8FI8FI-element formulation (cf. Figure 7 for the 20RI8FI-element formulation). Both formulations yield very similar results, indicating that the GND density field is not affected by the choice of element formulation.

**Figure 5:**
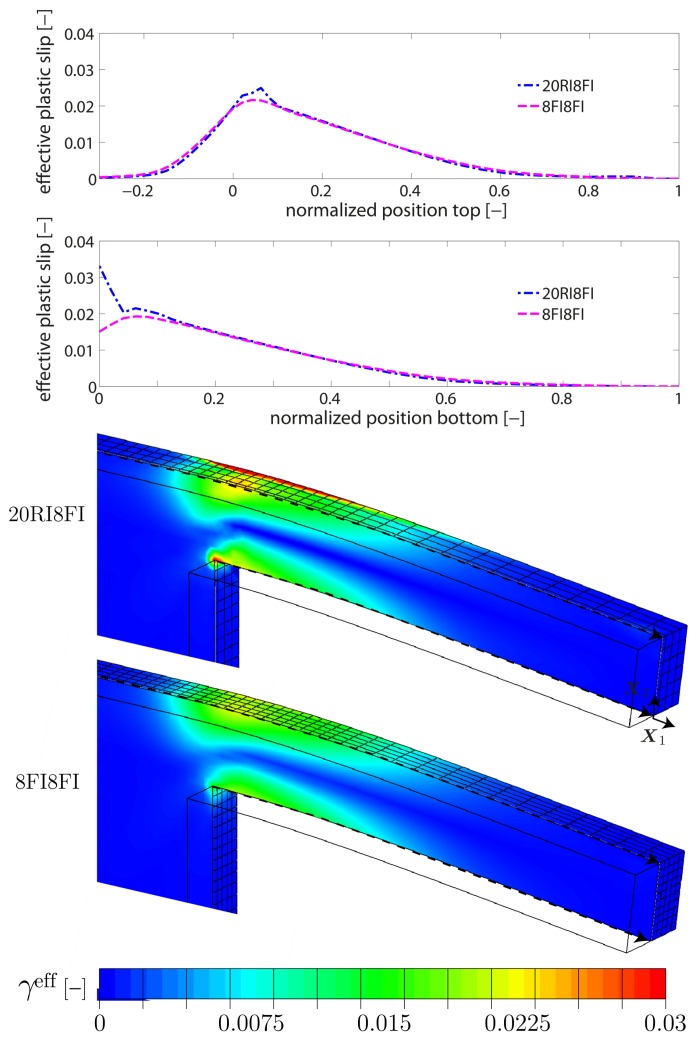
Distribution of the effective plastic slip $\gamma^{\mathrm{eff}}$ for the cantilever beam sample #1 along the indicated paths of the central cross section. In addition, the distribution is shown within the central $X_{1}-X_{3}$-plane. Results show some noticeable differences between both element formulations, in particular, close to the supported end, which refer mainly to the magnitude of $\gamma^{\mathrm{eff}}$.

In the following, a qualitative comparison with regard to the GND density and the plastic slip is carried out. For that reason, the effective GND density $g_{i}^{\mathrm{eff}}$ is introduced as a function of the admitted edge and screw GND components

(32) $$\begin{array}{c} g_{i}^{\mathrm{eff}}=\sqrt{\sum_{\alpha }{\left[g_{i\alpha }^{\mathrm{tot}}\right]}^{2}}=\sqrt{\sum_{\alpha }{\left[g_{i\alpha }^{e}\right]}^{2}+{\left[g_{i\alpha }^{s}\right]}^{2}}, \end{array}$$

where $g_{i\alpha }^{\mathrm{tot}}$ is the total GND density associated with slip system *α*. In analogy, the effective plastic slip

(33) $$\begin{array}{c} \gamma^{\mathrm{eff}}=\sqrt{\sum_{\alpha }\gamma_{\alpha }^{2}}, \end{array}$$

serves as a representative variable in order to compare the distribution of plastic deformation. Exemplarily, Figure 4 depicts the distribution of the effective GND density $g_{i}^{\mathrm{eff}}$ along the central middle axis for both element formulations. The results are within the same order of magnitude or even identical, i.e., the GND density field is well captured by both formulations. This holds true for both, the qualitative distribution along the middle central axis as well as the quantitative distribution within the $X_{1}-X_{3}$-plane. Consequently, the bending response (but not the GND evolution) is affected by volumetric locking in case of the 8FI8FI-element formulation. The contour plot for the case of 20RI8FI-elements is not presented as only marginal differences are present compared to the contour plot in Figure 4. Nevertheless, the contour plot of $g_{i}^{\mathrm{eff}}$ can be found in Figure 7 (Section 5.2) where the pile-up characteristic of GNDs is analyzed in more detail. The distribution of the effective plastic slip $\gamma^{\mathrm{eff}}$ is qualitatively compared along the highest and lowest central path parallel to the beam axis, see Figure 5. In addition, a quantitative comparison is provided on the basis of the effective plastic slip distribution within the central $X_{1}-X_{3}$-plane. As seen, differences are found near the supporting end, i.e., close to a normalized position of ≈0. Here, the 20RI8FI-element formulation resolves a higher magnitude of $\gamma^{\mathrm{eff}}$ which is confirmed by the contour plots. Slightly higher values of $\gamma^{\mathrm{eff}}$ are also computed in regions where both formulations are close to each other. Besides, it can be seen that plastic deformation is only accommodated within the first third/half of the beam sample (referred to the fixed side) whereas the rest of the beam finger remains straight. Such a localized plastic deformation is characteristic for cantilever beam bending.

### 5.2. Bending Size Effect—Influence of Sample Thickness

Experiments show that the bending size effect is strongly correlated to the beam thickness. For that reason, the ability of the model to predict a strengthening effect as a result of thickness reduction is studied. This investigation allows in turn to quantify the role of GNDs within cantilever beam bending experiments. All subsequent computations are based on the 20RI8FI-element formulation. All three sample sizes are loaded up to 10% normalized deflection using a gradient hardening modulus of $H_{0}^{e}=1$ GPa, which corresponds to $H_{0}^{s}=650$ MPa via Equation (22). A relatively high value is chosen in order to obtain a rather strong size effect such that the impact of the characteristic sample dimension is immediately recognizable. The length scale parameter *l* is involved in all subsequent computations with the value stated in Table 3.

**Figure 6:**
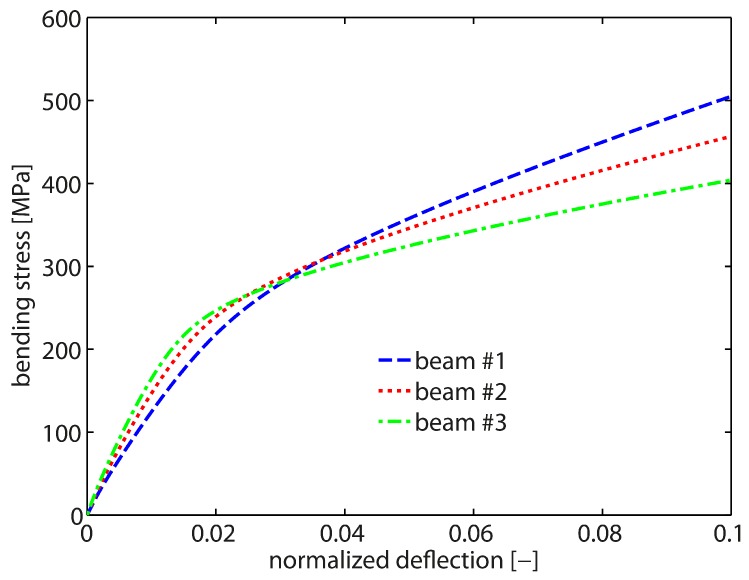
Bending response of cantilever beam samples with different thicknesses. The strengthening effect associated with the accumulation of GNDs is the strongest for cantilever beam #1, having the smallest thickness of t=2.5 μm. The softest response is obtained for cantilever beam #3 with t=5 μm. The general trend in terms of smaller is stronger is captured well by the underlying model. All computations refer to the 20RI8FI-element formulation.

The results in terms of stress–strain curves for all three cantilever beam samples are presented in Figure 6. A clearly recognizable increase in strength is obtained for all three beam sizes compared to the reference saturation stress, i.e., the size-independent response measured in [12]. The smallest sample (cantilever beam #1, thickness of t=2.5μm) exhibits the stiffest response. With increasing beam thickness, the bending response becomes softer. Accordingly, the response of cantilever beam #3 (thickness of t=5μm) is the softest and for t=3.5 μm (cantilever beam #2), the response is located in between. The obtained size effect is well captured by the non-local crystal plasticity model. The slop in the elasticity-dominated regime shows the exact opposite trend. Here, the slope is determined by the second moment of area and, hence, by the beam thickness *t*. At a later stage of deformation, the back-stress effect resulting from the storage of GNDs becomes dominant in terms of the work-hardening rate.

**Figure 7:**
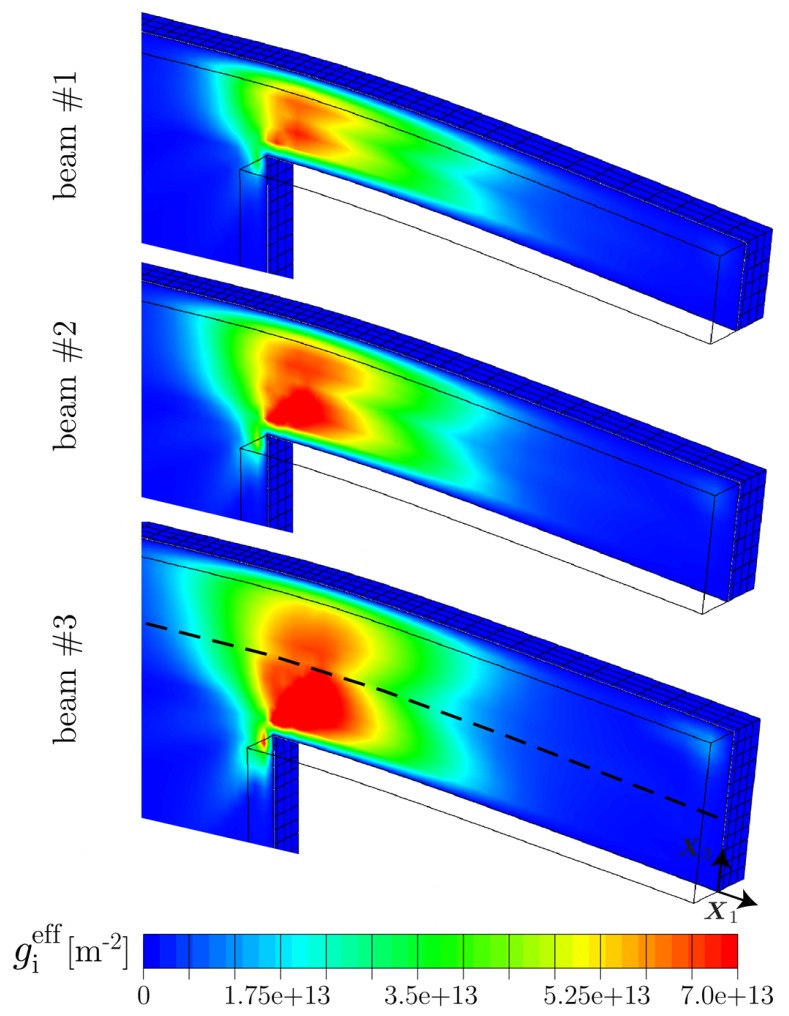
Distribution of the effective GND density $g_{i}^{\mathrm{eff}}$ for all three sample sizes within the central cross section ($X_{1}-X_{3}$-plane) after 10% normalized deflection. GNDs pile up along the neutral plane indicated by the dashed line. Although the distribution of $g_{i}^{\mathrm{eff}}$ appears to be similar with respect to all three beam sizes, their impact on the bending response is strongly correlated to the beam thickness via the plastic section modulus $S^{p}$. In view of the effective SSD density shown in Figure 8, a strong effect on the evolution is found due to the impact of GNDs.

By considering the distribution of GNDs within the central cross section of the beams, i.e., within the $X_{1}-X_{3}$-plane as displayed in Figure 7, it is seen that high densities of GNDs are accumulated at the supported end of the beams where the deformation is concentrated. In this region, GNDs pile up along the neutral plane (zero stress isoline), which does not necessarily coincide with the middle beam line. In fact, a shift of the stress field towards the bottom is caused to some extent by the supported end of the cantilever beam.

For all three sample sizes, a similar distribution of the effective GND density is found with a maximum value of $g_{i}^{\mathrm{eff}}\approx 7.0\times 10^{13}$ m${}^{-2}$, located at the lower half of the clamped side. In other words, the population of GNDs directly scales with the deformation gradient imposed by the normalized deflection as this is the same for all three beam geometries. Nevertheless, the resulting back-stress contributes differently for the samples leading to higher bending stresses $\sigma_{\mathrm{ben}}$ for thinner beam samples. This can be partially explained by the fact that the computed bending stress $\sigma_{\mathrm{ben}}=M_{b}^{p}/S^{p}$ depends on the plastic section modulus $S^{p}=wt^{2}/4$ which is a pure geometrical information. It is assumed that plastic deformation dominates which holds true at least in the most relevant strain regime $\varepsilon_{\mathrm{ben}}>$≈0.35, i.e., in the regime where the bending stress is saturating (cf. [12]). Further, $M_{b}^{p}=Fl_{b}$ denotes the plastic bending moment. Thus, the resulting flow stress scales inversely proportional to the square of the beam thickness *t*. Accordingly, there must be a scaling effect resulting from the beam width *w*, even if this effect is expected to be smaller. As *w* is kept constant in the present study, a pure dependence on the sample thickness is obtained. In the experiments, however, an influence of the beam width *w* is likely due to dimensional deviations caused by the fabrication process.

The role of GNDs is further assessed by means of the statistically stored dislocation density. To do so, we introduce the effective SSD density $\zeta_{i}^{\mathrm{eff}}$ as a function of $\gamma^{\mathrm{eff}}$ and the effective free path of moving dislocations $L^{\mathrm{eff}}$

(34) $$\begin{array}{c} {\dot{\zeta }}_{i}^{\mathrm{eff}}=\frac{\nu^{\mathrm{eff}}}{bL^{\mathrm{eff}}}, \end{array}$$

where

(35) $$\begin{array}{c} L^{\mathrm{eff}}=\frac{K}{\sqrt{\rho_{i}^{\mathrm{eff}}}}, \end{array}$$

$\rho_{i}^{\mathrm{eff}}=\zeta_{i}^{\mathrm{eff}}+g_{i}^{\mathrm{eff}}$ is the total (effective) dislocation density, and *K* is a material constant. Equation (34) represents a modified version of the originally proposed relation by Essmann and Mughrabi [40,41]. Corresponding interaction processes are intrinsically considered via the calculation of $\gamma_{\alpha }$ and $g_{i\alpha }$, respectively. K=10 ([42]) and $\zeta_{0}^{\mathrm{eff}}=2.0\times 10^{12}$ m${}^{-2}$ ([13]) are given in the literature.

**Figure 8:**
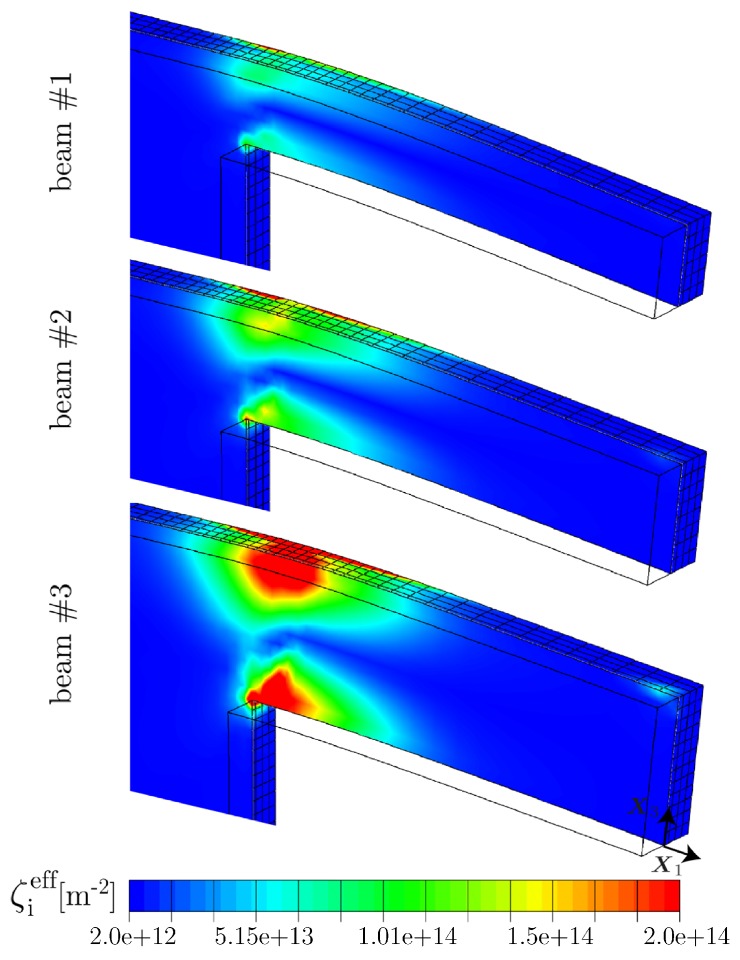
Distribution of the effective SSD density $\zeta_{i}^{\mathrm{eff}}$ for all three sample sizes within the central cross section ($X_{1}-X_{3}$-plane) after 10% normalized deflection. The density of SSDs approaches the density of GNDs with reducing beam thickness, leading to a more pronounced influence on the mechanical bending response coming from accumulated GNDs.

In Figure 8, the contour plots illustrate that the density of SSDs is naturally concentrated within highly deformed zones. More interestingly, the magnitude of $\zeta_{i}^{\mathrm{eff}}$ reduces considerably with decreasing thickness of the cantilever beam. This indicates that the impact of GNDs on the evolution of SSDs is increased for smaller samples. In other words, the magnitude of SSDs approaches the magnitude of GNDs with decreasing sample dimensions, leading to a more pronounced influence on the bending response in case of smaller beams coming from accumulated GNDs. This indicates that the location of most prominent dislocation accumulation, for instance in terms of the total dislocation density $\rho^{\mathrm{eff}}$, shifts from the beam surface towards the beam center with decreasing beam thickness.

### 5.3. Bending Size Effect—Impact of a Saturating GND Density

The plastic deformation is strongly localized at the supported end of the beam. In this region, the number of GNDs increases with increasing bending load due to compatibility reasons. Accordingly, the hardening contribution from the higher-order gradient ${\mathrm{Div}}_{i}\left(\kappa_{\alpha }\right)$ continuously increases which is reflected by the slope in the hardening behavior, cf. for instance Figure 6. However, from a physical point of view, the number of dislocations stored locally cannot be arbitrary high [43]. For that reason, the impact of a maximum permissible GND density on the bending response is investigated for cantilever beam #1 by setting up two different saturation values which are applied to each slip system independently: $g_{i\alpha }^{\max}=1\times 10^{13}$ m${}^{-2}$ and $g_{i\alpha }^{\max}=2\times 10^{13}$ m${}^{-2}$, respectively. Hence, the evolution equations for edge and screw GND densities are exposed to the following case differentiations:

(36) $$\begin{array}{c} {\dot{g}}_{i\alpha }^{e}=\left\{\begin{array}{cc} \mathrm{Equation}(14),\; & \mathrm{for}\;g_{i\alpha (n+1)}^{e}<g_{i\alpha }^{\max};\\ 0,\; & \mathrm{for}\;g_{i\alpha (n+1)}^{e}\geq g_{i\alpha }^{\max}, \end{array}\right. \end{array}$$

and

(37) $$\begin{array}{c} {\dot{g}}_{i\alpha }^{s}=\left\{\begin{array}{cc} \mathrm{Equation}(15),\; & \mathrm{for}\;g_{i\alpha (n+1)}^{s}<g_{i\alpha }^{\max};\\ 0,\; & \mathrm{for}\;g_{i\alpha (n+1)}^{s}\geq g_{i\alpha }^{\max}. \end{array}\right. \end{array}$$

**Figure 9:**
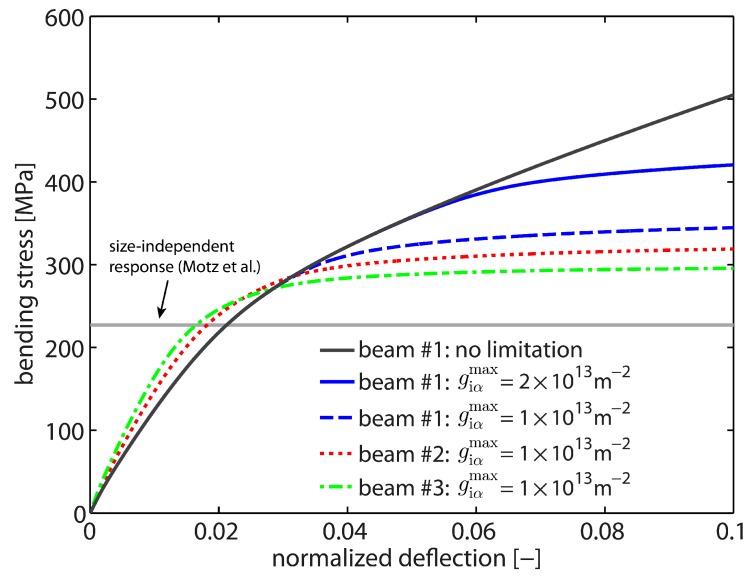
Impact of a maximum permissible GND density $g_{i\alpha }^{\max}$ on the mechanical bending response of Cu single crystals. As seen for cantilever beam # 1, the resulting saturation level is steered by the magnitude of $g_{i\alpha }^{\max}$. Furthermore, the bending size effect is conserved when comparing the response between the differently sized cantilever beams for a GND saturation limit of $g_{i\alpha }^{\max}=1\times 10^{13}$ m${}^{-2}$.

As in the previous cases, all computations are based on the 20RI8FI-element formulation and are performed up to 10% normalized deflection. The resulting stress–strain curves are presented in Figure 9 together with the size-independent reference and the response of an unrestricted GND density evolution. Those cases represent the lower resp. upper bound of the mechanical bending behavior in terms of strength. The bending response in case of a limited GND evolution shows a saturation-like hardening behavior where the increase in strength relative to the size-independent case is related to the applied GND density limit. Hence, the stress saturation level is steered by the magnitude of $g_{i\alpha }^{\max}$ in the way that a higher saturation limit causes a delayed deviation from the unrestricted case. In fact, once the saturation limit of $g_{i\alpha }^{\max}$ is reached, the size-dependent micro-hardening stresses become decoupled from the gradient of plastic slip. Accordingly the size-dependent hardening contribution coming from ${\mathrm{Div}}_{i}\left(\kappa_{\alpha }\right)$ is limited.

**Figure 10:**
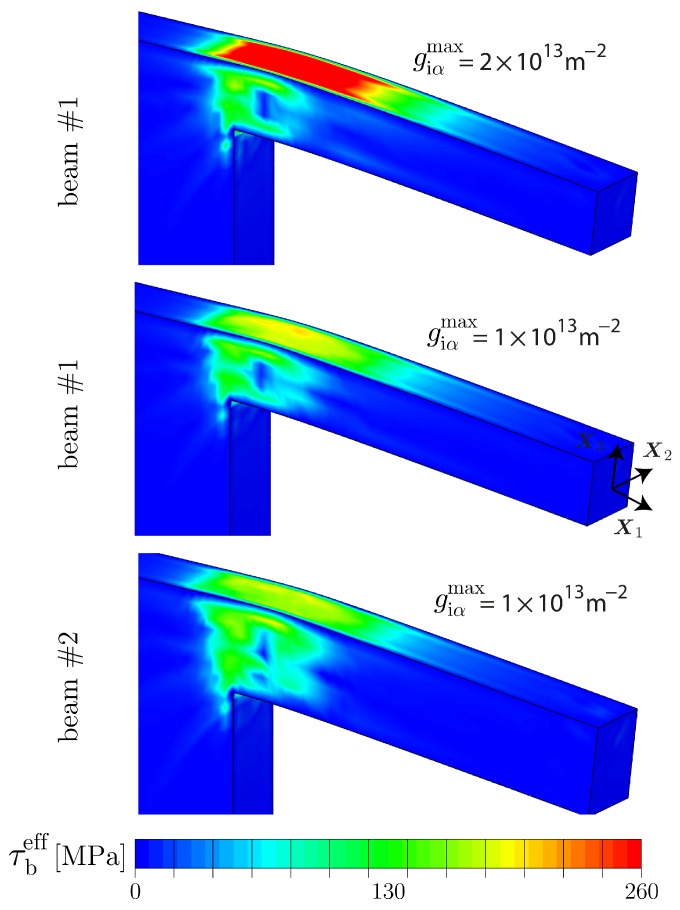
Effective back stress $\tau_{b}^{\mathrm{eff}}$ for selected $g_{i\alpha }^{\max}$ values and cantilever beam sizes. The size-dependent resistance to bending deformation increases with increasing GND density limit. The sample thickness has a negligible impact on the back-stress evolution for a fixed $g_{i\alpha }^{\max}$.

In related experiments, the bending response for a cantilever beam of size 2.5×5.0×16.3 μm ($t\times w\times l_{b}$) was found to show a stress saturation already at about 3.5% normalized deflection [12]. Thus, a saturation limit for GNDs around $1\times 10^{13}$ m${}^{-2}$ appears to be reasonable with respect to the current problem (cf. Figure 9). In comparison to the size-independent case, this yields an increase of about 118 MPa in flow stress which is solely related to the geometrically necessary storage of dislocations. Furthermore, it can be seen from Figure 9 that the bending size effect is conserved when comparing the bending response between the differently sized cantilever beams for the particular case if $g_{i\alpha }^{\max}=1\times 10^{13}$ m${}^{-2}$. The resulting effective back stress $\tau_{b}^{\mathrm{eff}}$, defined as

(38) $$\begin{array}{c} \tau_{b}^{\mathrm{eff}}=\sqrt{\sum_{\alpha }{\mathrm{Div}}_{i}{\left(\kappa_{\alpha }\right)}^{2}}, \end{array}$$

is compared in Figure 10 for selected magnitudes of $g_{i\alpha }^{\max}$ and sample thicknesses *t*. As can be seen, the size-dependent hardening contribution is much higher for the higher GND density limit which is consistent with the determined bending response in Figure 9. At the same time, the impact of the thickness is negligible for a particular $g_{i\alpha }^{\max}$ value.

## 6. Discussion

The bending size effect is characterized by an increasing strength with decreasing sample thickness. A strong correlation between flow stress and beam thickness was found by Motz et al. [12] for thicknesses in the range 7.5 μm to 1 μm. A similar trend was found by Demir et al. [26] for alternative single crystal geometries with average thicknesses in the range 4.23 μm to 1.02 μm. In this size regime, the bending size effect is associated with a combination of different mechanism. Besides the geometrically necessary storage of dislocations, dislocation starvation and dislocation source limitation are known to affect the plastic deformation behavior. Dislocation source limitation plays a dominant role for very small beam sizes as the statistical distribution of dislocation sources becomes then more and more crucial, cf. [44]. As a consequence of a limited availability of source density within the localized region of plastic deformation (supported beam end), the yield limit of the material may increase significantly. In this respect, the storage of GNDs imposes an additional resistance to dislocation nucleation, yielding an increasing nucleation strength. Last but not least, plasticity is strongly controlled by the initial dislocation density of the crystal [8]. In the particular case of microbending of single crystals, dislocations are able to leave the crystal through the free surfaces at some point [12]. This process of starving or escaping dislocations finally leads to the extinction of initially available dislocations [4]. Hence, with a sufficiently small number of obstacles in the single crystalline sample, the size-dependent flow stress is additionally governed by the applied deformation rate relative to the dislocation nucleation rate which determines the required stress level for continuing operation of individual dislocation sources, see also Balint et al. [45]. Keeping in mind that an accurate fabrication of micro-cantilever samples is a challenging task, there is currently no sufficient data available allowing for a meaningful interpretation of the impact of each of the sample dimensions—thickness, width, and length—independently. For that reason, strengthening effects mentioned above are neglected in the current numerical study. Instead, the focus is solely on the micromechanical role of GNDs and their impact on the mechanical bending response as a function of the beam thickness *t*.

**Figure 11:**
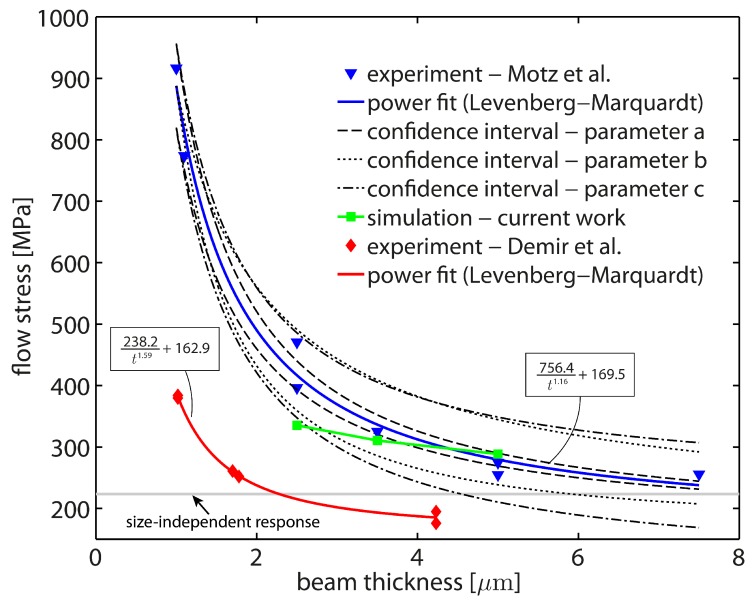
Experimentally and numerically determined relation between flow stress $\sigma_{\mathrm{ben}}$ and beam sample thickness *t* in microbending of Cu single crystals together with associated power fits. The deviations between the two experimental data sets might be explained by the different sample geometries: cantilever beams with rectangular cross section and high $l_{b}/t$ as well as $l_{b}/w$ ratios ([12]); cantilever beams with trapezoidal cross section, high $l_{b}/t_{\mathrm{avg}}$ ratios, but low $l_{b}/w$ ratios ([26]). The numerically determined flow stress values show a reasonable strengthening effect in the regime where GNDs dominate the micromechanical behavior of the crystal, i.e., in the range t≳3 μm. For t≲3 μm, the impact of dislocation starvation and source limitation become crucial, leading to an even more pronounced increase in flow stress with decreasing beam thickness *t*. The data of Demir et al. refers to a flow stress measurement at 0.06 strain.

The numerical results correctly capture the commonly observed trend ‘smaller is stronger’. In fact, the size-dependent strengthening effect due to the back-stress effect induced by the storage of GNDs is predicted very well for two different cases: (i) unrestricted evolution of GND densities and (ii) physically limited GND densities. In case (i), GND densities evolve with increasing plastic slip gradients which, for the particular case of cantilever beam bending, continuously grow within the deformation-localized region. In contrast, the evolution of GND densities is limited in case (ii) and completely vanishes if a certain saturation limit is reached, i.e., if the plastic strain gradient becomes very large. This mimics a more realistic micromechanical behavior as is supported by the characteristics of related experimental force–deflection curves. Moreover, the local number of GNDs to be stored locally cannot be arbitrary high (cf. [43]). The numerically determined flow stresses for the here investigated beam thicknesses and for the particular case of $g_{i\alpha }^{\max}=1\times 10^{13}$ m${}^{-2}$ are illustrated in Figure 11 along with available experimental data from the literature. The simulation data is solely a function of the beam thickness *t* while experimentally determined data sets might (undesired) be affected by the reduction of the other sample dimensions ($l_{b}$ and *w*) or by varying dimension ratios ($l_{b}/t$ and $l_{b}/w$). Apparently, changing sample dimensions and their ratios add substantially to the complexity of the superimposed effect associated with the mechanisms discussed above. This might be one explanation for the deviations between the experimental data sets of [12,26]. A meaningful discussion regarding the impact of the initially available dislocation density is not possible due to the lack of data.

Both experimental data sets were fitted by a power function of the form $at^{-b}+c$ using the Levenberg–Marquardt algorithm, see Figure 11. Then, an error analysis is conducted for the data set of Motz et al. [12] in order to draw final conclusions regarding the statistical representativeness of the measurements. The standard deviation is calculated for each fitting parameter *a*, *b*, and *c*. The maximal and minimal standard deviation of each fitting parameter is considered independently to compute upper and lower confidence bounds while the other two parameters are kept constant in each case. The resulting confidence intervals are embedded in Figure 11 and are interpreted independently. The overall fit sensitivity associated with the prefactor *a* is small due to its narrow confidence interval over the entire beam thickness range. With respect to the exponent *b*, it is found that the power function is governed by the exponent *b* in the regime t<2μm. As here, one would expect rather large experimental scatter, the power fit appears less sensitive compared to the regime t>2μm. In fact, the confidence interval associated with the exponent *b* vanishes if *t* approaches ≈1 μm. Hence, we conclude that the determined exponent describes the experimentally measured relation between $\sigma_{\mathrm{ben}}$ and *t* fairly well. The offset parameter *c* indicates a similar tendency as the exponent, i.e., for t>2μm the power function becomes more sensitive with respect to *c*. In comparison to the simulation data which reflects a pure dependence on the beam thickness *t*, a good agreement is found for t≳3μm as the predicted data lies very close to the actual fit. Consequently, it is concluded that the mechanism associated with the geometrically necessary storage of dislocations governs the mechanical bending behavior in this range. For t≲3μm, the impact of dislocation starvation and source limitation becomes obviously non-negligible. These findings fit also to DDD predictions of Hussein et al. [46] where single crystals with D≤1.0 μm were found to be almost free of dislocations due to the limiting size whereas crystals with D≥5.0 μm show pronounced dislocation activities.

## 7. Conclusions

Based on an extended gradient crystal plasticity model, the role of GNDs in the mechanical bending response of micron-sized, single crystalline Cu was investigated. The underlying model contains non-standard evolution relations for the edge and screw components of the slip system-based dislocation density vector, crystal-specific interaction relations, and higher-order gradient boundary conditions. The cantilever beam geometries considered in the numerical study allowed the examination of the strengthening effect associated with the geometrically necessary storage of dislocations solely as a function of the beam thickness which is a non-trivial task from an experimental point of view. Other size-dependent mechanisms such as dislocation starvation and source limitation were disregarded at the current stage. In particular, the influence of the beam thickness as well as the impact of a maximum permissible GND density was of primary importance. Besides, in relation to the coupled field problem between displacement and GND density degrees of freedom, a quantitative comparison between two different finite element formulations has been carried out. On the basis of our findings, we conclude:

- The bending dominated deformation is captured more accurately by the mixed FE-formulation denoted as 20RI8FI. In contrast, the commonly applied linear FE-formulation (8FI8FI) overestimates the bending response for the size-independent as well as the size-dependent case. The locking phenomenon only influences the predicted bending behavior (and not the predicted GND density) in the case of the 8FI8FI-element formulation.
- The bending size effect is captured by the theory to the extent caused by geometrically necessary storage of dislocations. This size-dependent strengthening effect can be explained as follows: (i) Similar dislocation pile-ups have been found around the neutral plane where dislocations get stuck rapidly and lose the ability to accommodate the beam bending, independent of the beam size. The impact of the resulting back-stress effect on the bending response is nevertheless higher for the smallest beam as the bending stress is inversely proportional to the square of the beam thickness. The same holds for the flow stress computation in related cantilever beam bending experiments; (ii) In contrast to the distribution of the GND density, a much higher population of SSDs was found for the largest cantilever beam sample which indicates that the bending behavior is here mainly governed by random trapping processes. However, with decreasing beam thickness, these processes become less pronounced. This is supported by the fact that the magnitude of the SSD density becomes comparable to the one of the GND density in the case of the thinnest beam sample. Consequently, the impact of GNDs on the mechanical bending response is most pronounced in the thinnest beam sample. Accordingly, the location of maximum dislocation storage was found to shift from the sample surface towards the beam center when decreasing the beam thickness.
- A physically motivated limitation of the GND density was incorporated into the model by modified evolution equations for the edge and screw GND density components. In the current crystal plasticity framework, this was done at the nodal level as GND densities were treated as additional degrees of freedom. This leads to a bending response with saturation-like hardening behavior—which is in accordance with experimental findings. At the same time, the smaller is stronger trend was conserved in accordance to the unrestricted case. In the end, a saturation limit of ≈1 $\times 10^{13}$ m${}^{-2}$ was found to match well the characteristics in the bending response of related experimental data where a flow stress saturation was obtained at about 3.5% normalized deflection.
- Numerically determined flow stresses using a saturation limit of ≈$1\times 10^{13}$ m${}^{-2}$ show a reasonable strengthening effect in the beam thickness range t≳3 μm. The predicted flow stress of cantilever beam #3 is in great accordance with experimental data. The flow stress associated with cantilever beam #2 still shows an acceptable accuracy as it lies within the confidence interval of the related experimental data. For the thinnest beam sample, a considerable contribution from another size-dependent mechanism occurs. Based on this, it can be argued that GNDs dominate the micromechanical bending response in the thickness range t≳3 while other mechanisms such as dislocation starvation and source limitation become crucial for t≲3 μm where an even more pronounced increase in flow stress is experimentally measured.

## Figures and Tables

**Table 1:** Sample dimensions and discretization data for the here investigated cantilever beam geometries.

Beam	$l_{b}$ [μm]	*w* [μm]	*t* [μm]	Elements
#1 (8FI8FI)	15	2.5	2.5	8790
#1 (20RI8FI)	15	2.5	2.5	3864
#2 (20RI8FI)	15	2.5	3.5	4480
#3 (20RI8FI)	15	2.5	5.0	5712

**Table 2:** C.s.s. of copper (fcc): slip direction $s_{\alpha }$, transversal slip direction $t_{\alpha }$, slip plane normal $n_{\alpha }$, and initial Schmid factor $f_{\alpha }$. Slip activation is mainly determined by the bending stresses as an accommodation of plastic deformation by slip systems with an initially zero Schmid factor is rather unlikely.

$\sqrt{2}s_{\alpha }$	$\sqrt{6}t_{\alpha }$	$\sqrt{3}n_{\alpha }$	$f_{\alpha }$
$\left[01\bar{1}\right]$	[211]	$\left[1\bar{1}\bar{1}\right]$	0.4082
[101]	$\left[\bar{1}\bar{2}1\right]$	$\left[1\bar{1}\bar{1}\right]$	0.4082
$\left[\bar{1}\bar{1}0\right]$	$\left[\bar{1}1\bar{2}\right]$	$\left[1\bar{1}\bar{1}\right]$	0
$\left[0\bar{1}1\right]$	$\left[2\bar{1}\bar{1}\right]$	[111]	0
$\left[10\bar{1}\right]$	$\left[\bar{1}2\bar{1}\right]$	[111]	0
$\left[\bar{1}10\right]$	$\left[\bar{1}\bar{1}2\right]$	[111]	0
[011]	$\left[\bar{2}1\bar{1}\right]$	$\left[\bar{1}\bar{1}1\right]$	0
$\left[\bar{1}0\bar{1}\right]$	$\left[1\bar{2}\bar{1}\right]$	$\left[\bar{1}\bar{1}1\right]$	0
$\left[1\bar{1}0\right]$	[112]	$\left[\bar{1}\bar{1}1\right]$	0
$\left[0\bar{1}\bar{1}\right]$	$\left[\bar{2}\bar{1}1\right]$	$\left[\bar{1}1\bar{1}\right]$	0.4082
$\left[\bar{1}01\right]$	[121]	$\left[\bar{1}1\bar{1}\right]$	0.4082
[110]	$\left[1\bar{1}\bar{2}\right]$	$\left[\bar{1}1\bar{1}\right]$	0

**Table 3:** Material parameters of Cu single crystal used for numerical computations.

Young’s modulus	*E*	126.9	GPa
Poisson’s ratio	*ν*	0.35	-
Microscopic yield stress	$Y_{\alpha }$	1.5	MPa
Local hardening modulus	$H_{0}^{l}$	77.5	MPa
Gradient hardening modulus	$H_{0}^{e}$	1	GPa
Saturation rate	$c_{\mathrm{sat}}$	$10^{3}$	-
Reference slip rate	$\nu_{0}$	10${}^{-3}$	s^-1^
Rate sensitivity parameter	*m*	20.0	-
Drag stress	$C_{0}$	10.0	MPa
Length scale	*l*	4.0	μm
Length of Burgers vector	*b*	0.2552	nm

